# Biographic Clinics

**Published:** 1907-09

**Authors:** 


**Biographic Clinics.** By George M. Gould, M.D. Vol. III. Pp. 516. London: Rebman Limited. 1905.

Dr. Gould starts on the assumption that scarcely any recognition is given by physicians, or even by oculists, to the fact that errors of refraction give rise to numerous discomforts, which might be removed by the application of suitable spectacles ; and if the gravamen were true that the medical profession does not fairly consider the conditions of eye-strain, and their bearings on the treatment of migraine and other numerous discomforts, his book would be of service in drawing attention to the undoubted and well-recognised fact that the correction of astigmatism and of refraction errors is frequently essential for the relief of numerous patients, and very useful in helping many others.

But he seems to assume that he is preaching a new gospel, while on the contrary he is overstating and exaggerating the value of a well recognised and very widely applied form of treatment. He says: "The Continent of Europe still lingers in pitiful barbarism upon this subject. When my patients return from these benighted countries, they tell tales that should be gathered for the amusement of coming and humour-loving generations." "Patients suffering from eye-strain are the prey of the travelling spectacle vendor, who should often be jailed, the quack optician and oculist, the jeweller, who knows nothing of optics or of ophthalmology."

At any rate, there seem numerous efforts being made to apply the remedy which Dr. Gould appears to consider the one panacea for all human illness. But we can assure him that refraction testing is well understood, and is applied with every scientific care by English oculists and by their colleagues throughout the Continent of Europe, and that the book before us is not likely to be of the least service in assisting the investigation of eye-strain from any scientific or clinical or unique standpoint. The book is amusing, and may give hope even to sufferers from incurable maladies, that they may yet be relieved by what is arrogantly called the "new ophthalmology."

No intelligent ophthalmic surgeon will find any help in the book, except to encourage him to plod on with refraction errors, with the certainty that some good will accrue to his patient from the use of spectacles. The nasal surgeon is informed that errors of refraction cause sinus disease. He will naturally retort, and with good reason: "No, it is the sinus disease that caused the eye symptoms, which you will in vain try to relieve by spectacles."

Migraine is no doubt very often due to eye-strain—everyone has known this for many years—and English physicians always send such cases to the oculist before they attempt any treatment by advice or drugs. It is unfair to the practitioner to say that eye-strain is not always sought for as a cause of migraine ; it is unfair for any writer of recent years to claim any originality for suggesting the use of glasses in these cases. A narrow specialist, an exaggerator who has become an extremist, has indeed become a hobby rider in the writing of this book (p. 38).

John Addington Symonds is supposed to have suffered from an uncorrected eye-strain, to which alone all his pains and weaknesses are attributed ; but there is no positive evidence adduced of any error of refraction or of muscle balance.

Taine's ill-health is attributed by Dr. Gould to a strabismus and ptosis that he suffered from. But there are plenty of squinters and those with paralysed eye muscles who do not suffer on that account from any other defect of health. Eye-strain is often a cause of headache, and may cause other neuroses. But many neuroses exist, and headache may be present, without ocular defects. The first inquiry in migraine should be as to the eye conditions ; but migraine may be present without abnormality of eye, and the correction of eye defect may be quite inoperative against migraine symptoms.

The later chapters in the book are interesting, and show considerable knowledge and investigation. Dr. Gould quotes in support of his views writers of articles chiefly in 1904 and 1903. But as long ago as 1893, an article insisting on the necessity for careful testing of the eyesight and the conditions of the muscle balance in asthenopia and ocular headache, &c., appeared in the *Bristol Medico-Chirurgical Journal*. Writers in this *Journal* have supported the view that careful refraction work and the proper adaptation of glasses is an essential form of medical diagnosis and treatment ; but they do not claim spectacles as an infallible cure for all forms of ill-health.

